# *Agrobacterium*-Mediated Transient Expression Methods to Validate Gene Functions in Strawberry (*F.* × *ananassa*)

**DOI:** 10.3390/plants13233290

**Published:** 2024-11-22

**Authors:** Yoon Jeong Jang, Hyeondae Han, Seonghee Lee

**Affiliations:** 1 Vegetable Research Division, National Institute of Horticultural and Herbal Science, Rural Development Administration, Wanju 55365, Republic of Korea; jangyj770@korea.kr (Y.J.J.); hhd0916@korea.kr (H.H.); 2 Horticultural Sciences Department, IFAS Gulf Coast Research and Education Center, University of Florida, Wimauma, FL 33598, USA

**Keywords:** transient assays, *Agrobacterium*-mediated, agroinfiltration, octoploid strawberry

## Abstract

Understanding gene function is important for crop improvement and breeding efforts, especially in a genetically complex polyploid plant species such as the octoploid strawberry. *Agrobacterium*-mediated transient assays are a widely used tool for investigating gene functions and offer a reliable alternative to stable transformation. However, variability in tissue-specific responses and inconsistent applicability of *Agrobacterium*-mediated transient assay across diverse plant species can be challenging. In this study, we provide a method utilizing *Agrobacterium*-mediated transient expression to examine the function of genes in octoploid strawberry. Our approach encompasses leaf, root, and fruit tissues, providing a comprehensive strategy for validating gene functions in strawberries. Through meticulous optimization and validation in planta, this method offers valuable insights into gene function in strawberry functional genomics and genetics research. By addressing tissue-specific variability, our methodology serves as a valuable technical resource that could facilitate advancements in identifying gene functions in octoploid strawberry.

## 1. Introduction

Recent advancements in genomic research and the development of high-quality genome assemblies for octoploid strawberry have significantly enhanced our ability to understand gene functions. This progress has led to an increased demand for functional genomics research proposed to improve strawberry traits like disease resistance and fruit quality. Traditionally, gene functions in plants were studied using methods like RNA interference (RNAi)-mediated gene silencing and overexpression. However, in recent years, CRISPR-Cas9 technology has become a powerful tool for gene function validation and crop improvement [1]. Despite the potential of *Agrobacterium* transformation and tissue culture for identifying gene functions, it remains time-consuming, labor-intensive, and not universally applicable to all plant species, especially fruit crops [2]. For instance, the production of transgenic strawberry plants through the standard tissue culture process can take 20–30 weeks [3]. Therefore, to accelerate gene function studies, an efficient protocol for *Agrobacterium*-mediated transient assays is essential, allowing for the rapid identification of key genes that can be used in crop improvement efforts.

Transient assay offers a faster, scalable method for producing recombinant proteins and validating target gene functions compared with the lengthy process of creating transgenic plants. One of the key advantages of transient expression is that foreign DNA, specifically the T-DNA copy, does not integrate into the host genome. Instead, it utilizes *Agrobacterium* to infiltrate the plant and generate a significantly larger number of T-DNA copies within the plant cell [4,5,6]. These unintegrated gene fragments remain transcriptionally active for several days, allowing gene expression to be examined as early as 3 days and up to 10 days after inoculation [7,8]. Although transient expression levels gradually decrease over time due to the instability of the non-integrated T-DNA copies, they offer a rapid and efficient approach to functional genomics studies. On the contrary, stable T-DNA integration results in a substantial increase in gene expression over time [9].

Several studies have reported successful applications of transient assays in strawberry leaves and fruits [10,11,12]. In strawberry fruits, transient assays have been conducted using genes related to fruit development and the flavonoid biosynthetic pathway [10,11,13]. Hoffmann et al. (2006) demonstrated gene function validation in octoploid strawberries by knocking down the ripening-related chalcone synthase (CHS) gene via an RNAi vector. This study revealed that reduced CHS mRNA levels corresponded with decreased enzymatic CHS activity [11]. In another study by Pi et al. (2019), the anthocyanin synthesis master regulator gene *FveMYB10* was overexpressed in diploid strawberries using attached fruits. The overexpression of fruits led to the accumulation of red pigments in the white-fruited Yellow Wonder (YW). Additionally, in octoploid strawberry, functional analysis of the Reduced Anthocyanin in Petioles (RAP) gene was conducted using the RNAi vector on the ‘Sweet Charlie’ cultivar, resulting in a significant reduction in anthocyanin accumulation 1-week post-inoculation [10]. More recently, Zhao et al. (2019) conducted a transient assay study on both detached and attached strawberry fruits, assessing optimal conditions for *Agrobacterium*-mediated gene expression. Using reporter genes eGFP and GUS under the 35CaMV promoter, they determined that full injection in detached fruits yielded the best results. Optimal assay conditions included an incubation temperature of 20–25 °C and 4–6 days post-inoculation, with fruits at the Large to White stage showing the most efficient expression [14].

For the leaf detached transient assay, a rapid and efficient *Agrobacterium*-mediated transient gene expression system was optimized for strawberry leaves to facilitate gene function analysis, particularly in disease resistance studies [12]. The target gene, the broad-spectrum disease resistance protein *RPW8.2*, was transiently expressed in strawberry leaves. Additionally, *Agrobacterium*-transfected leaves were treated with exogenous salicylic acid (SA) and inoculated with the strawberry powdery mildew pathogen *Podosphaera aphanis*, which led to increased expression of the *RPW8.2* protein. The findings suggest that constitutive expression of *RPW8.2* plays a role in regulating defense-related genes, contributing to enhanced resistance mechanisms in strawberry leaves [12]. In this study, a range of parameters was evaluated and optimized, including plant culture conditions, strawberry cultivars, seedling age, leaf position, and various physical and chemical factors influencing the culture medium used for vacuum infiltration of transfected leaves. Our *Agrobacterium*-mediated transient gene expression method can be effectively used for validating gene functions in the leaf, fruit, crown, and root tissues of strawberry. Despite these advances, no transient assays have yet been reported for root and crown tissues, even in diploid strawberries, highlighting a methodological gap for these vital critical structures.

*Agrobacterium*-mediated transient transformation has been widely used for functional studies in many plant species, but effective protocols for conducting transient assays in octoploid strawberry are still lacking. In this study, we developed a protocol for *Agrobacterium*-mediated transient assays specifically for octoploid cultivated strawberry. This method provides a valuable resource for rapidly investigating the functions of genes associated with disease resistance and fruit quality and could facilitate functional genomics research into strawberry.

## 2. Results

**Figure 1:**
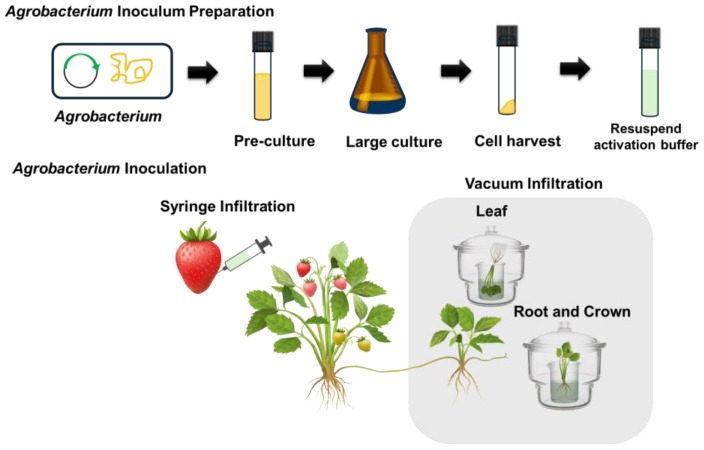
A schematic diagram illustrating the transient assay process. The top section outlines the growth process of *Agrobacterium*, including pre-culture and large-scale culture, followed by the harvesting of *Agrobacterium* cells and resuspension in an activation buffer. A schematic diagram illustrating the inoculation methods for each strawberry tissue. For fruit, *Agrobacterium* infiltration is performed using a syringe, while for leaf, root, and crown tissues, vacuum infiltration is employed.

Our aim in this research was to optimize the *Agrobacterium*-mediated transient assay across various strawberry tissue, including fruit, leaf, and crown (with root). We first transformed a gene of interest into *Agrobacterium*. After transformation, the *Agrobacterium* was initially grown in a small culture, followed by confirmation of the transformation via colony PCR. Once confirmed, the transformed *Agrobacterium* was transferred to a fresh liquid medium for large-scale culture. The cultured transformed *Agrobacterium* was harvested and resuspended in an activation buffer containing 200 µM acetosyringone and incubated for 3 h in dark room temperature conditions. The prepared inoculum was then utilized for functional gene assays: syringe infiltration was applied to fruits, while vacuum infiltration was conducted for leaves and crown (root) tissues (Figure 1).

### 2.1. Fruit Transient Assay

**Figure 2:**
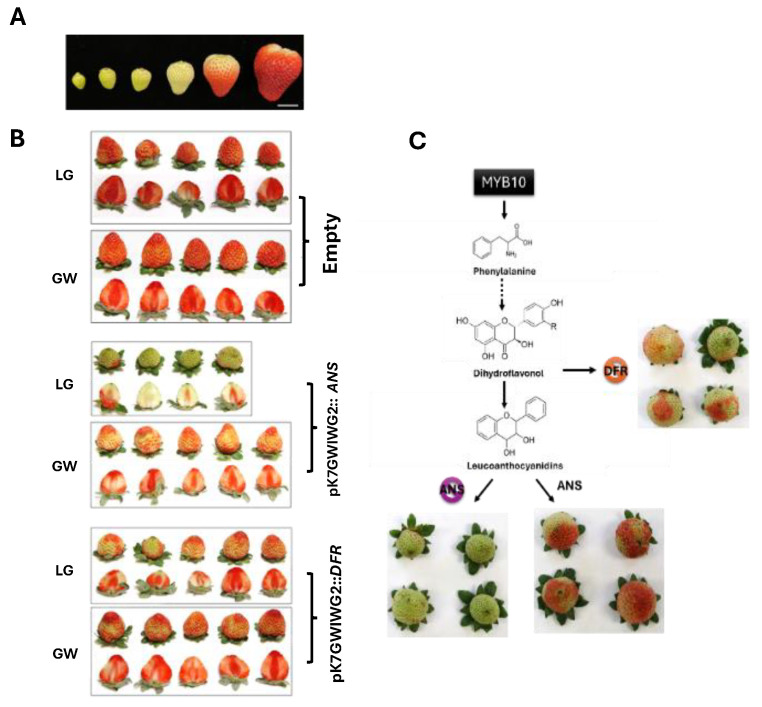
RNAi-mediated transient assay endogene silencing of ANS and DFR in strawberry fruits. (**A**) Developmental stages of the cultivar ‘Florida Brilliance’ (*Fragaria* × *ananassa*): small green (SG), medium green (MG), large green (LG), greenish-white (GW), turning red (TR), and red (R). Scale bar indicates 2 cm. (**B**) The phenotypes of fruits agroinfiltrated with pK7GWIWG2 (empty vector), pK7GWIWG2::*ANS*, or pK7GWIWG2::*DFR* are shown. (**C**) This figure illustrates the confirmation of stage-specific silencing at the LG and GW stages, targeting two genes, *ANS* and *DFR*, involved in the anthocyanin biosynthetic pathway. The dotted arrows represent the consecutive steps in the pathway from phenylalanine to dihydroflavonol, while solid arrows indicate direct transitions to the subsequent steps from dihydroflavonol.

In this study, the transient assay in fruit targeted two genes involved in the anthocyanin biosynthetic pathway, which is responsible for the synthesis of anthocyanins through a series of catalytic reactions. As a case study in the fruit transient assay, we investigated the biological functions of anthocyanin synthase (ANS) and dihydroflavonol 4-reductase (DFR), which are crucial enzymes in the pathway converting dihydroflavonol into leucoanthocyanidin [15,16]. This assay was conducted at the large green (LG) and greenish-white (GW) stage of strawberry fruit development. An intriguing observation was made regarding the differential effects of RNAi on anthocyanin biosynthesis between the *ANS* and *DFR* genes within the same developmental fruit stage. Notably, the RNAi-mediated silencing of *ANS* showed a pronounced impact on anthocyanin synthesis, which was more evident than in *DFR*. Specifically, in the LG stage, RNAi-mediated knockdown of *ANS* significantly delayed anthocyanin production compared with the GW stage, where the effect was less pronounced (Figure 2).

### 2.2. Leaf Transient Assay

A *Pestalotiopsis*-like fungus causing severe fruit rot and leaf spots has emerged in commercial fields in Florida, resulting in significant plant losses. Since its appearance, the disease has remained a major problem, affecting at least 80 ha of strawberry fields and greenhouse strawberries for the first time [17,18]. Unfortunately, all commercially grown strawberry varieties are highly susceptible to this pathogen, and the genetic mechanisms for resistance have yet to be fully understood.

**Figure 3:**
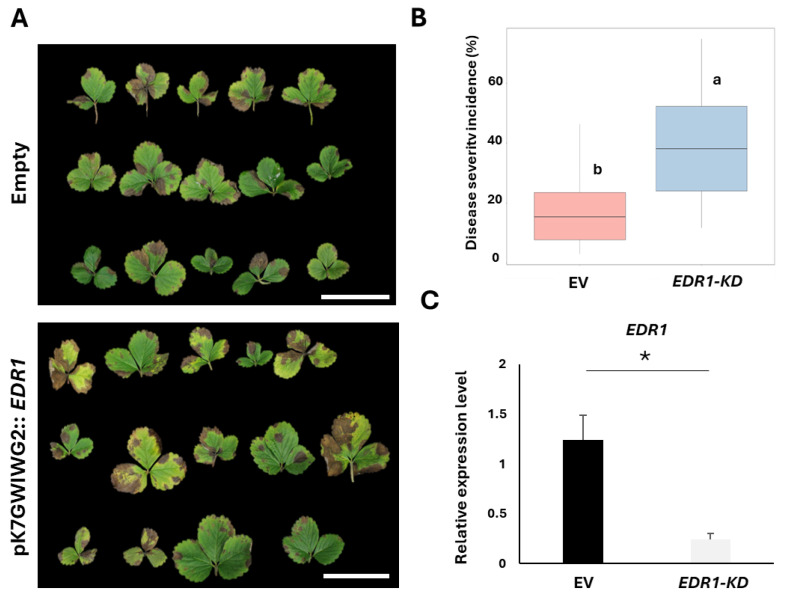
RNAi-mediated transient knockdown of EDR1 in strawberry leaves. (**A**) RNAi-mediated knockdown transient leaf assay and disease phenotypes after infection of *Neopestalotiopsis* spp. at 7 days post-inoculation (dpi): pK7GWIWG2 empty vector and pK7GWIWG2::*EDR1*. Scale bar: 2 cm. (**B**) Disease severity incidence (%) in strawberry leaves from RNAi experiments was quantified using ImageJ software (version 1.54 g) for image data analysis. Statistical comparisons were conducted with one-way ANOVA followed by Tukey’s HSD post hoc test to assess treatment means, with significance denoted by different letters (‘a’ and ‘b’) for groups (*p* ≤ 0.05). The X-axis represents *Agrobacterium*-transfected plants, including pK7GWIWG2 (EV) and pK7GWIWG2::*EDR1* (*EDR1*-KD), while the Y-axis shows disease severity incidence (%). (**C**) qRT-PCR analysis was performed to evaluate the gene expression of EDR1 in RNAi-mediated knockdown plants compared with control plants (EV). The X-axis represents *Agrobacterium*-transfected plants, including EV and *EDR1*-KD, while the Y-axis indicates the relative expression level. An asterisk (*) denotes a significant difference according to the standard deviation test: * *p* < 0.05.

In this study, we employed a reverse genetics strategy to identify genes linked to resistance in T-DNA-tagged Arabidopsis mutants. Through a search of public databases and relevant literature, we identified several candidate genes potentially involved in fungal resistance, including *AtEDR1* (CS67959, AT1G08720), *AtMYB46* (SALK_088514C, AT5G12870), *AtMPK3-1* (SALK_151594, AT3G45640), *AtMYB46* (SALK_100993C, AT5G12870.1), *AtDMR6* (SALK_203382C, AT5G24530), *AtNPR3* and *AtNPR4* (CS72352, AT5G45110, AT4G19660), *AtWRKY70* (SALK_025198C, AT3G56400), and *AtWRKY53* (SALK_034157C, AT4G23810). Mutant lines for these genes were sourced from the Arabidopsis Information Resource (TAIR, https://www.arabidopsis.org) and subsequently inoculated with *Neopestalotiopsis* spp. (Figure 3A and Figure S1, and Table S1). As a result, we identified several genes involved in resistance to the pathogen *Neopestalotiopsis* spp. Notably, the *edr1* (*ENHANCED DISEASE RESISTANCE 1*) mutant showed high susceptibility to the pathogen, which makes it an ideal candidate for validating our transient assay system in strawberry. We cloned the *EDR1* homolog in the strawberry genome and applied transient RNAi assays to confirm the role of *EDR1* in strawberry against *Neopestalotiopsis* spp. It was found that RNAi-mediated *EDR1*-knockdown plants are more susceptible compared with the control plants (empty vector) (Figure 3A,B and Table S2). Additionally, qRT-PCR analysis confirmed that the gene expression level of *EDR1* in the knockdown plants was approximately 6-fold lower than in the control (Figure 3C and Table S3).

### 2.3. Crown and Root Transient Assay

**Figure 4:**
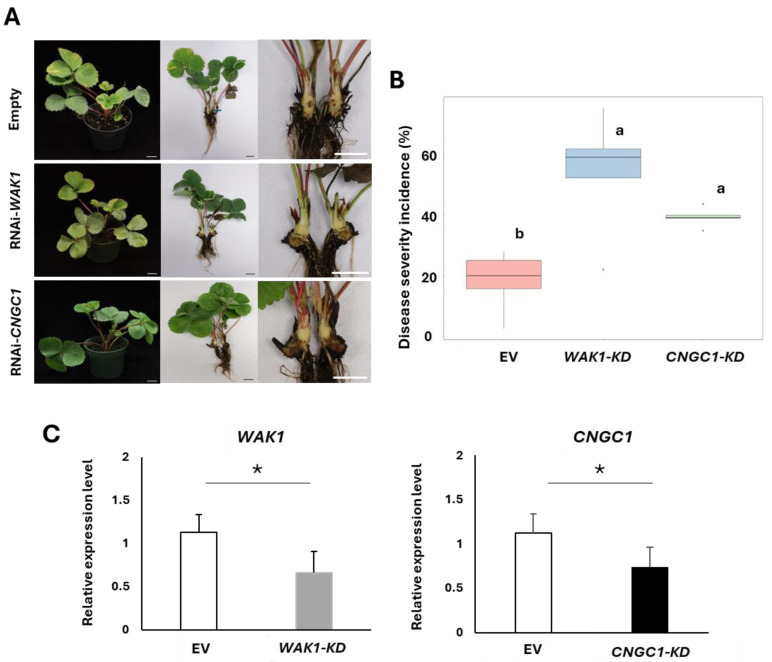
RNAi-mediated transient knockdown of *WAK1* and *CNGC1* in strawberry crown and root. (**A**) Transient crown and root assay showing *Phytophthora cactorum* infection 17 days post-inoculation (dpi) in strawberry crowns infiltrated with RNAi constructs of *WAK1*, *CNGC1*, and an empty vector control. Scale bar: 2 cm. (**B**) Disease severity incidence (%) in strawberry crown and root assay from RNAi experiments was quantified using ImageJ software for image data analysis. Statistical comparisons were conducted with one-way ANOVA followed by Tukey’s HSD post hoc test to assess treatment means, with significance denoted by different letters (‘a’ and ‘b’) for groups (*p* ≤ 0.05). The X-axis represents *Agrobacterium*-transfected plants, pK7GWIWG2 (EV), pK7GWIWG2::*WAK1* (*WAK1*-KD), and pK7GWIWG2::*CNGC1* (*CNGC1*-KD), while the Y-axis indicates disease severity incidence (%). (**C**) qRT-PCR analysis was performed to determine the gene expression of *WAK1* and *CNGC1* in RNAi-mediated knockdown and control plants (EV). The X-axis represents *Agrobacterium*-transfected plants, EV, *WAK1*-KD, and *CNGC1*-KD, while the Y-axis indicates the relative expression level. An asterisk (*) denotes a significant difference according to the standard deviation test: * *p* < 0.05.

Phytophthora crown rot (PhCR), caused by *Phytophthora cactorum*, is among the most devastating soilborne diseases impacting strawberries both in the United States and worldwide [19]. In a previous study, we identified the *Resistance to Phytophthora cactorum 2* (*RPc2*) locus through QTL mapping and transcriptome analysis, which revealed the candidate genes *Wall Associated Kinase 1* (*WAK1*) and *Cyclic Nucleotide Gated Channel 1* (*CNGC1*) [20]. Using this information, we inoculated the resistant cultivar ‘Fronteras’ [21] to validate the function of genes associated with PhCR resistance. In this study, we conducted a transient assay on resistant plants to examine the phenotype after inoculation with RNAi-cloned *Agrobacterium*. As expected, the empty vector showed no noticeable disease symptoms; the only observed marks were localized to the syringe-wounded areas on the crown, with no further symptom of pathogenic infection. The empty vector displayed a Disease severity incidence (DSI) of 25.2%, whereas plants with *WAK1*-Knockdown (KD) and *CNGC1*-Knockdown (KD) constructs showed increased DSIs of 61.9% and 40%, respectively, which represents a 2.4-fold and 1.5-fold increases in infection lesion area compared with the control. Furthermore, plants expressing the *WAK1*-KD and *CNGC1*-KD constructs exhibited more severe disease symptoms, including wilting and crown rot, which were significantly more severe compared with the empty vector controls. This indicates the functional role of these genes involved in the resistance to PhCR (Figure 4A,B and Table S2). Additionally, qRT-PCR analysis showed that the expression levels of *WAK1* and *CNGC1* showed about a 1.7 and 1.6-fold reduction in RNAi-mediated knockdown plants, respectively (Figure 4C and Table S3).

## 3. Discussion

In this study, we successfully developed an efficient *Agrobacterium*-mediated transient assay system for octoploid strawberry, covering various tissues, including fruit, leaf, crown, and root. Functional gene validation is especially important in the context of CRISPR-Cas9 gene editing, which is increasingly used for precise plant breeding and crop improvement [22]. Identifying key genes quickly is essential for efficiently applying CRISPR-based gene editing, as it allows for the targeted improvement of traits of interest. The octoploid genome of cultivated strawberry presents additional challenges in gene function studies, as homoeologous genes may exist in multiple copies, leading to gene redundancy or complex expression patterns. In conducting the RNAi-mediated transient assay, we focused on achieving broad targeting across all subgenomes due to the complexity associated with homoeologous gene copies, which contributes to gene redundancy.

While transient assays in strawberry fruit and leaf have been demonstrated in previous studies [11,12,16,23], methods for transient expression in other tissues have not yet been explored. For fruit assays, Zhao et al. (2019) established optimal parameters for transient gene validation in octoploid strawberry, comparing both attached and detached fruit methods. Their study showed that detached fruits, incubated at 25 °C for 4–6 days post-inoculation, achieved maximum GFP and GUS expression at the Large to White developmental stages of fruit. Attached fruit assays, involving *Agrobacterium*-mediated transfection while the fruit remains on the plant, maintain the fruit’s natural physiological environment, allowing for a more accurate representation of ripening processes due to the continuous supply of nutrients and hormones. However, consistent environmental control across multiple fruits in this setup is challenging, as the method is influenced by whole-plant variability, which detached fruit assays cannot replicate [10,14]. In contrast, detached fruit assays, where fruits are separated from the plant prior to transformation, provide a controlled and manageable environment beneficial for high-throughput studies, as treatments can be applied uniformly across numerous samples. Nonetheless, detachment can alter the physiological responses of the fruit, potentially impacting gene expression results [23,24]. It has been shown that the developmental stage of the fruit is also crucial in transient gene expression assays. Greenish-white strawberries are particularly suitable due to their permeable structure, which facilitates effective *Agrobacterium* infiltration and allows for early-stage ripening processes to be studied without the added complexity of full maturation. At this stage, the fruit’s cellular structure is mature enough to support infiltration with minimal structural damage while remaining physiologically stable to ensure consistent and reliable results [13]. Expanding on these findings, we performed transient assays in strawberry fruits using an RNAi vector targeting the ANS and DFR genes, critical enzymes involved in converting dihydroflavonol to leucoanthocyanidin [15,16]. Specifically, at the LG stage, ANS knockdown significantly delayed anthocyanin production compared with the GW stage, identifying the LG stage as the most effective for transient assay analysis across the six developmental stages examined (Figure 2).

For leaf assays, Cui et al. (2017) optimized an *Agrobacterium*-mediated transient gene expression system for detached strawberry leaves to express the broad-spectrum disease resistance protein RPW8.2, focusing on factors such as plant culture conditions, strawberry cultivar, seedling age, leaf position, and various physical and chemical properties of the culture medium used in vacuum infiltration [12]. Their study found that leaves from the first leaf position of in vitro plantlets at various seedling ages exhibited the highest YFP signal. However, preparing plant material for transient assays in octoploid strawberries can be time-intensive, and propagated plants and meristem-derived plants require 13 weeks to develop [25], while strawberry tissue regeneration through traditional tissue culture can take approximately 6 months [26]. By contrast, transient expression assays in this study offer a faster, more efficient alternative, enabling gene function analysis within days. To overcome time limitations, this study utilized 3–4-week-old runner-propagated plants for leaf transient assays, providing a more practical approach.

Notably, transient assays in strawberry root and crown tissues have not been reported previously. For instance, strawberry leaf, crown, and root tissues are a lot more challenging to infiltrate *Agrobacterium*-containing vector constructs than fruits. To address these challenges, our study explored a combination of vacuum infiltration, agro-drench soaking, and needle wounding, drawing from previous research by Levy et al. (2005) and Burman, N. et al. (2020), which demonstrated effective transient gene expression in Arabidopsis thaliana roots and rice seedling through *Agrobacterium*-mediated transformation [27,28]. This approach utilizes hydroponically grown Arabidopsis roots for subcellular localization, enabling precise tracking of GFP fusion proteins [27]. Similarly, in rice, the use of surfactants in conjunction with wounding and vacuum infiltration significantly improves transformation efficiency by overcoming natural barriers in the leaf structure, as observed in rice leaves where GUS staining exhibited prominent expression in roots, coleoptiles, and leaves. Although root visualization can be challenging due to their morphology, they yielded a satisfactory frequency of GFP expression, underscoring the potential of roots as viable targets for transient assays alongside leaf tissues [28]. Our approach yielded effective GUS expression in strawberry roots, as confirmed by staining results (Figure S2) that showed robust GUS gene activity. This finding highlights the feasibility of using root tissues for transient assays despite the inherent challenges in infiltrating these tissues. The success of this method can be attributed to the strategic combination of vacuum infiltration, agro-drench soaking, and needle wounding, which collectively helped to overcome natural barriers in the root structure and significantly boosted transformation efficiency. By leveraging these techniques, our study expands the application of transient assays to root tissues, aligning with similar successes observed in other systems, such as rice, where wounding and surfactant use also enhance gene delivery to root and coleoptile tissues. This approach underscores that with efficient protocol adaptations, roots can be effective targets for transient gene expression studies.

In conclusion, the development of this *Agrobacterium*-mediated transient assay system across multiple strawberry tissues offers a powerful and efficient platform for gene function validation. Recent advancements in high-quality haplotype-phased genome assemblies and genomics research have enabled the identification of genes related to important breeding traits in strawberry. The ability to rapidly validate these genes across different strawberry tissues provides crucial insights into trait development and significantly enhances our capacity to improve crop performance through functional genomics research.

## 4. Materials and Methods

### 4.1. Plant Materials

For fruit assays, ‘Florida Brilliance’ [29] plants were used. The developmental stage of the fruit plays a crucial role in the success of the transient assay [30]. In this study, we utilized Florida Brilliance [29] daughter plants for leaf-attached transient assays and ‘Fronteras’ non-UF cultivated plants for crown and root transient assays. To prepare for leaf-attached, crown, and root transient assays, daughter plants were propagated via runners, a process that should be initiated 3–4 weeks prior to the transient analysis. To apply reverse genetics for screening resistance to *Neopestalotiopsis* spp., we took the homozygous Arabidopsis T-DNA knockout lines (Clo-0 ecotype as a control, CS67959, SALK_088514C, SALK_151594, SALK_100993C, SALK_203382C, CS72352, SALK_025198C, SALK_034157C), which were sourced from TAIR (https://www.arabidopsis.org) (Table S1). Arabidopsis seeds underwent surface sterilization with 70% ethanol, followed by rinsing with sterilized water three times. The seeds were then sown on half-strength Murashige and Skoog (MS) medium containing 1.5% sucrose (*w*/*v*), and cultivated at 22 °C under a 16 h light/8 h dark cycle. After germination, seedlings were transplanted to soil and maintained at 25 °C with a 14 h light period before being used for inoculation tests.

### 4.2. Gene Cloning and Vector Construction

Given the challenges associated with amplifying subgenome-specific targeted genes in strawberry using conventional PCR, we synthesized gene fragments ranging from 300 to 350 bp through Twist Bioscience (San Francisko, CA, USA). To validate gene function via a transient assay, we constructed an RNAi vector using pK7GWIWG2. Initially, the gene fragments were cloned into the pDONR207 vector (Thermo Fisher, Waltham, MA, USA) through a BP reaction utilizing Gateway^®^ BP Clonase^TM^ II (Thermo Fisher, Cat. 11789-100) according to the manufacturer’s protocol. The cloned fragments were subsequently verified through colony PCR and sequencing before being inserted into the silencing RNAi Gateway^®^ vector pK7GWIWG2 to use LR reaction (Thermo Fisher Cat. 11791-020). Successful transformants were confirmed by PCR and sequencing.

Following sequencing to verify hairpin structures, the plasmid vector was transferred into EHA105 *Agrobacterium* cells via electroporation. Post-electroporation, 700 μL of LB broth medium (without antibiotics) was added to the transformed cells, which were then incubated for 1 h at 28 °C in a shaking incubator. The recovered transformed *Agrobacterium* cells were spread out onto the LB broth plate medium containing appropriate antibiotics. Selective LB broth plate medium was incubated at 28 °C for 2 days. A single *Agrobacterium* colony was put into 5 mL of LB broth liquid medium containing the appropriate antibiotics in a 15 mL tube and incubated at 28 °C in a shaking incubator at 220 rpm overnight. Colony PCR was performed using cultured *Agrobacterium* and a positive control plasmid vector. A large culture of LB broth liquid medium was performed using *Agrobacterium* identified through colony PCR as the final inoculum.

### 4.3. Preparation of Plant Tissues

For the transient expression assay, fruits were collected at two developmental stages: large green and light greenish-white, with 20 fruits per stage. The assay was employed to validate the function of candidate genes at each developmental stage. Fruits were collected and stored on ice to maintain freshness. After a gentle rinse with tap water, the fruits were immersed in a 0.7% sodium hypochlorite solution for 6 min, with gentle rotation to ensure even sterilization. Following this, the fruits were rinsed four times with sterilized distilled water, patted dry with sterilized paper towels, and placed in plastic containers to air dry in a clean hood for 20 min. Finally, the fruits were uniformly placed on an egg shelf.

To validate candidate gene function at each developmental stage, leaves from five 3–4-week-old runner plants were collected. For leaf sample preparation, prior to inoculation, any dust or soil was removed from the plants by gently brushing it off with a soft brush. If the leaves were wet, they were carefully dried with sterilized tissue to prevent dilution of the inoculum concentration.

To verify the function of candidate genes through a transient assay in roots and crown tissues, the study was conducted using 6 plants with an empty vector and 5 plants each for two genes of interest as replicates. When preparing roots, it is essential to carefully brush off any soil, ensuring that the roots remain intact and undamaged. Following soil removal, the roots were thoroughly rinsed with tap water. To prevent desiccation before inoculation, the roots were wrapped in sterilized tissue soaked in sterilized water, ensuring the tissue was adequately moist.

### 4.4. Preparation of Agrobacterium Inoculum

For the primary culture, a single colony was picked and inoculated into 5 mL of LB broth liquid medium supplemented with the appropriate antibiotics. The culture was incubated in a shaking incubator at 28 °C and 220 rpm, in dark conditions, overnight. For the revived culture, 1 mL of the primary culture was put into 400 mL of LB liquid medium supplemented with the necessary antibiotics, 10 mM MES (pH 5.6), and 20 μM acetosyringon. The mixture was allowed to incubate overnight in a shaking incubator set at 28 °C and 220 rpm, under dark conditions, until OD_600_ reached 0.6–0.8. Following overnight incubation, the *Agrobacterium* cells were harvested by centrifugation at 4000 rpm for 20 min at room temperature. After discarding the supernatant, the cells were resuspended in an activation buffer (10 mM MES, 10 mM MgCl_2_, and 200 μM acetosyringone), adjusting the OD600 to 0.5 for root inoculation and 0.8 for leaf and fruit inoculations. The optical density of the resuspended cells was measured, considering the final volume of the inoculation solution, to ensure accurate preparation. The final *Agrobacterium* inoculum, with 200 μM acetosyringon added according to the volume of the inoculation solution, was incubated at room temperature with gentle agitation at 70 rpm for 3–4 h prior to use.

### 4.5. Leaf Agroinfiltration

Prior to the vacuum infiltration step, Silwet-77 was added to the *Agrobacterium* inoculum at a final concentration of 0.05%. We ensured that the leaves were completely submerged in the *Agrobacterium* solution within the beaker. To inoculate the leaves, the plants were inverted to ensure they were fully immersed in the inoculation solution (Figure 1). The vacuum pump was activated for 3 min and then turned off. With the knob still closed, the vacuum was gradually released by gently opening the valve, ensuring a slow release to prevent damage to the plant tissue. We confirmed that the tissue was uniformly infiltrated; if any part of the leaf was not fully infiltrated with the solution, the infiltration process was repeated. After inoculation, the humidity around the plants was maintained to prevent desiccation. The plants were then incubated in a plastic container under dark conditions for 2 days, followed by a transition under 16 h of daylight and 8 h of darkness at room temperature.

### 4.6. Root Agroinfiltration

Prior to initiating the vacuum infiltration, Silwet-77 was added to the *Agrobacterium* suspension at a final concentration of 0.05%, ensuring that the roots were fully immersed in the solution within the beaker. Before applying the inoculum to the roots and crown, the areas were lightly scored and wounded using a syringe needle to facilitate entry of the *Agrobacterium*. For the inoculation of roots and crowns, the plants were positioned upright so that their roots were completely submerged in the inoculum. The vacuum pump was activated for 5 min, then deactivated, and the valve closed. The plants were allowed to remain submerged in the inoculation solution for an additional 5 min (Figure 1). With the valve still closed, the vacuum was gradually released to avoid causing damage to the plant tissue. After vacuum infiltration, the plants were left in the inoculation solution for 3 h at room temperature in the dark. Following this incubation, the plants were transferred to 4-inch pots filled with soil. Approximately 10 mL of the *Agrobacterium* solution was applied to the soil as a drench. To prevent desiccation post-inoculation, the plants were maintained in a controlled environment with humidity. They were then incubated in darkness for 2 days, followed by exposure to light under a 16 h light/8 h dark photoperiod.

### 4.7. Fruit Agroinfiltration

The fruits were arranged on egg shelves, ensuring an even distribution of large and small fruit across all shelves. Using a 3 mL sterile syringe, the *Agrobacterium* suspension was injected into both the upper and lower portions of each fruit. As the *Agrobacterium* solution was introduced, the fruit’s surface absorbed the solution, resulting in a translucent appearance. The control fruit was then inoculated first to reduce the risk of cross-contamination. Between each handling, gloves were changed and the workspace disinfected to further mitigate contamination risks. The inoculated fruit samples were placed on the egg shelves in a plastic box. The box was lined with two layers of sterile, moistened paper towels to maintain adequate humidity. The box was secured with its lid, and the samples were incubated in darkness for 2 days. Following this, the samples were transferred to light conditions with a 16 h light/8 h dark cycle.

### 4.8. Pathogen Preparation and Inoculation

To validate the target genes in different plant tissues, such as leaves and crowns, we employed specific pathogen preparation and inoculation techniques. For the leaves, the inoculation protocol followed the method outlined by Alam et al. (2024) for *Neopestalotiopsis* pathogens [31]. For the crown, we adhered to the inoculation and *Phytophthora cactorum* spore concentration methods, as described in a prior study [32]. Four days after *Agrobacterium*-mediated transformation, pathogens were inoculated using techniques tailored to each specific pathogen. Post-inoculation, humidity was maintained at optimal levels to promote pathogen growth and successful infection.

### 4.9. RNA Sampling and Extraction

Four days post-*Agrobacterium* inoculation, RNA sampling was conducted for both the control and treatment groups. For fruit samples, vertical sections were taken, avoiding the apex and base, with each sample consisting of 100 mg of tissue ground into a fine powder using liquid nitrogen. Leaf samples ranged from 40 to 60 mg, while root samples weighed approximately 30 mg. All samples were ground to a fine powder using liquid nitrogen and a mortar and pestle. Total RNA was isolated from the leaf, fruit, and crown tissues following the protocol outlined in the Spectrum™ Plant Total RNA Kit (Sigma-Aldrich, St. Louis, MO, USA). Post-extraction, RNA purity was assessed using a Nanodrop spectrophotometer, ensuring a 260/280 ratio between 1.8 and 2.2. Quantification of RNA was then performed. To eliminate residual DNA, 1 μg of total RNA was treated with Deoxyribonuclease I (DNase I) (Invitrogen, Carlsbad, CA, USA), following the manufacturer’s protocol. The DNase I-treated RNA was reverse transcribed into cDNA using oligo(dT) primers and M-MLV reverse transcriptase (M0253, New England BioLabs, Ipswich, MA, USA).

### 4.10. Quantitative PCR Analysis

Primers for housekeeping genes and target genes were designed using Primer3 software [33]. Quantitative PCR (qRT-PCR) was conducted using LightCycler^®^ 480 system (Roche Diagnostics, Mannheim, Germany) and Forget-Me-Not™ EvaGreen^®^ qPCR Master Mix (Biotium, CA, USA), according to the manufacturer’s instructions. PCR conditions were set as follows: an initial denaturation at 95 °C for 5 min, followed by 40 cycles of 95 °C for 10 s, annealing at 60 °C for 10 s, and extension at 72 °C for 10 s. Relative gene expression was determined using the ΔCq method, where the quantification cycle (Cq) value of the target gene was subtracted from the Cq of *FaGAPDH*. The log fold change was then calculated using the 2−ΔΔCq method [34].

### 4.11. Phenotype Validation

To quantify the symptomatic areas on the leaf and crown tissues, ImageJ software was employed to measure the regions exhibiting characteristic symptoms. Statistical analysis was then performed using R software (version 4.3.3), where a one-way ANOVA was conducted. Tukey’s Honestly Significant Difference (HSD) test was applied to compare the means across all treatments, ensuring the statistical significance of the findings (*p* ≤ 0.05).

## Supplementary Materials

The following supporting information can be downloaded at: https://www.mdpi.com/article/10.3390/plants13233290/s1, Figure S1: Phenotypic responses to *Neopestalotiopsis* spp. in *Arabidopsis* mutants. The X-axis represents *Arabidopsis* T-DNA knockout lines, while the Y-axis shows disease severity incidence (%); Figure S2: GUS histochemical assay of transient assay strawberry; Table S1: List of fungi-related associated gene *Arabidopsis* T-DNA knockout lines; Table S2: ANOVA results for pairwise comparisons of mean values among RNAi-treated and control groups; Table S3: Results of qRT-PCR analysis showing pairwise *t*-test comparisons of mean expression levels between RNAi-treated and empty control groups.
