# Supplementary material for: Agrobacterium-Mediated Transient Expression Methods to Validate Gene Functions in Strawberry (F. × ananassa)
Source: Plants (Basel). 2024 Nov 22;13(23):3290. doi: 10.3390/plants13233290 (PMC11644488; doi:10.3390/plants13233290)
Supplement: Supplementary file 1 [file plants-13-03290-s001.zip › Spp. Fig.pdf]

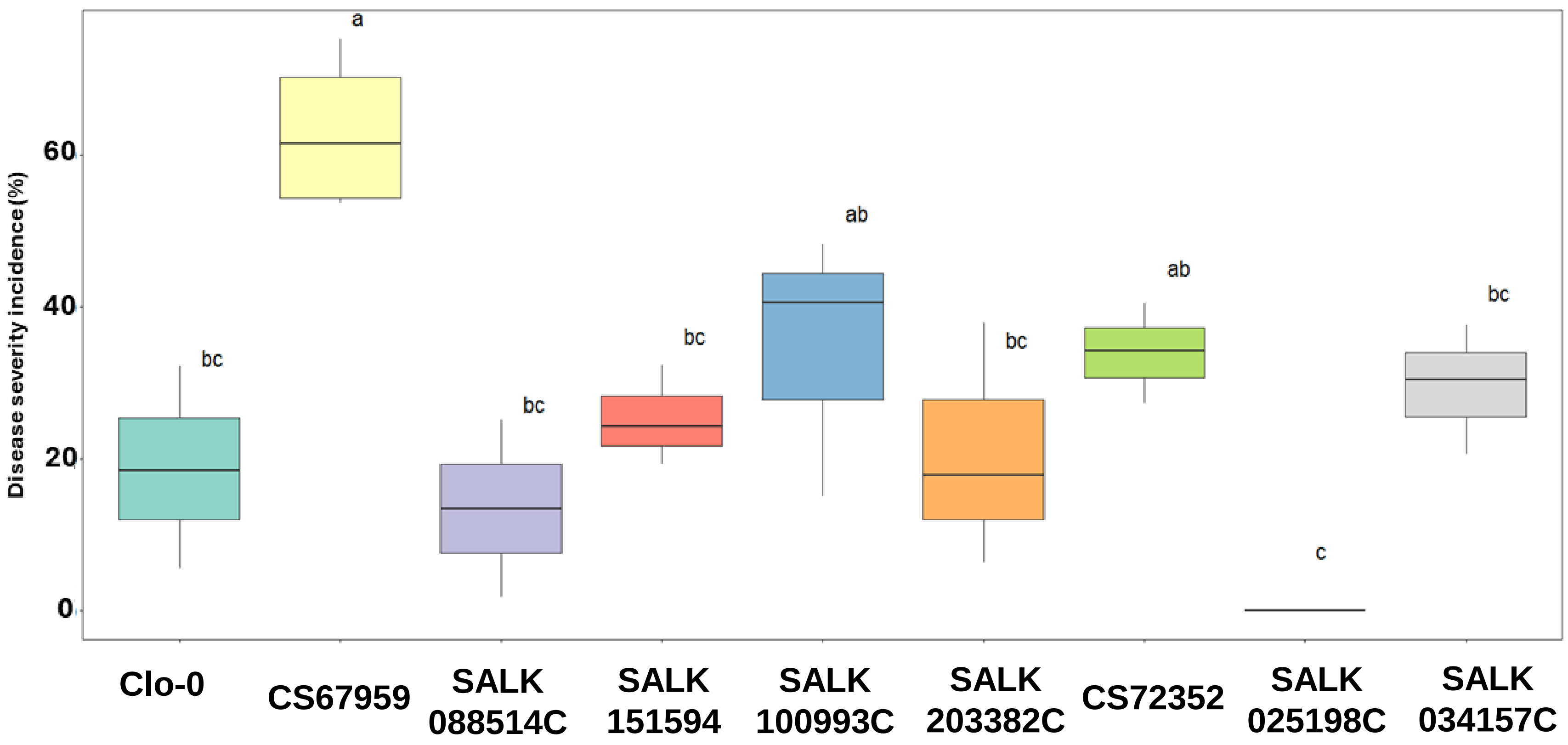

Supplementary Figure S1. Phenotypic responses to *Neopestalotiopsis* spp. in *Arabidopsis* mutants. The X-axis represents *Arabidopsis* T-DNA knockout lines, while the Y-axis shows disease severity incidence (%).

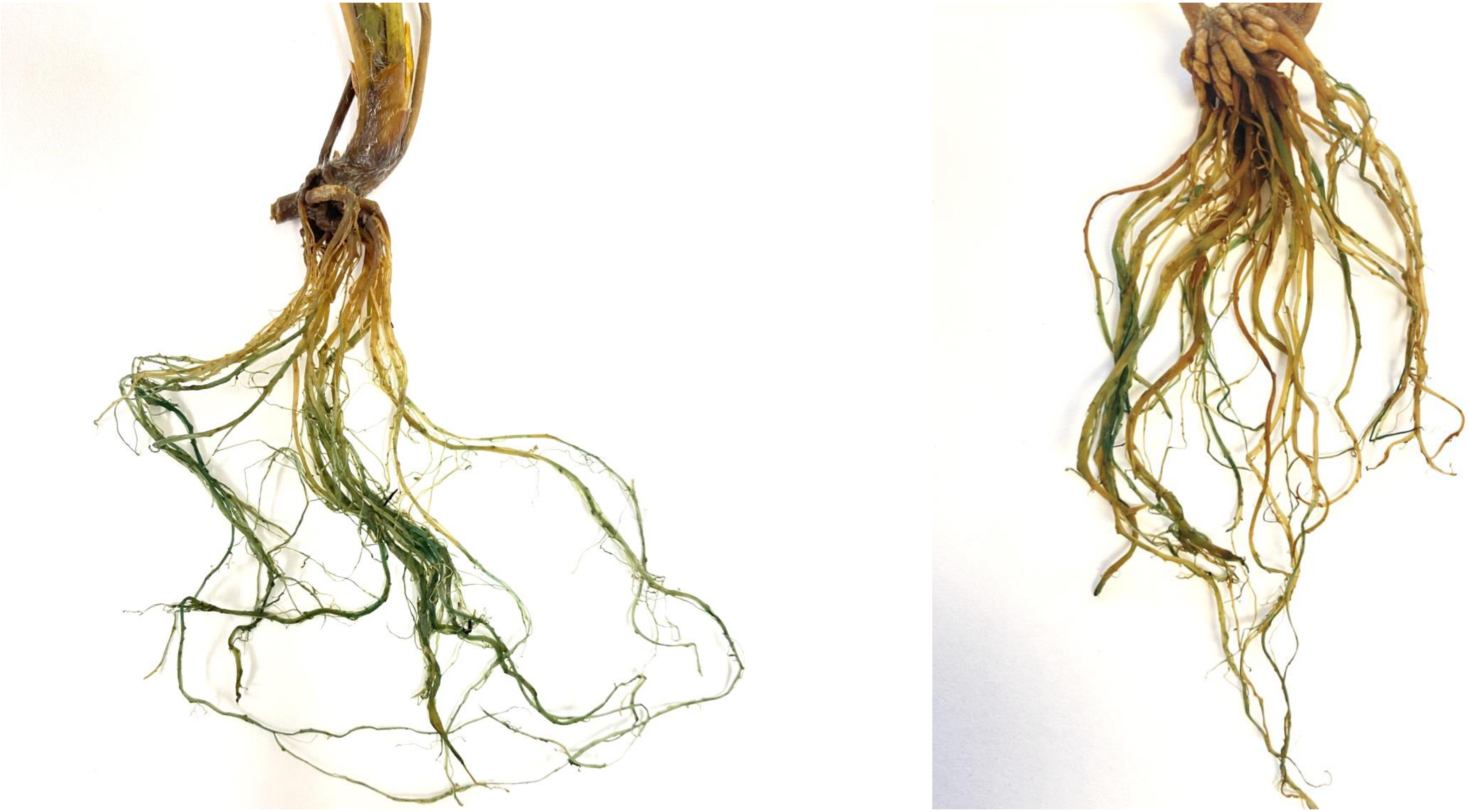

Supplementary Figure S2. GUS histochemical assay of transient assay strawberry.
