# Supplementary material for: Agrobacterium-Mediated Transient Expression Methods to Validate Gene Functions in Strawberry (F. × ananassa)
Source: Plants (Basel). 2024 Nov 22;13(23):3290. doi: 10.3390/plants13233290 (PMC11644488; doi:10.3390/plants13233290)
Supplement: Supplementary file 1 [file plants-13-03290-s001.zip › Supplementary Tables_final.pdf]

Supplementary Table S1. List of fungi-related associated gene Arabidopsis T-DNA knockout lines.

Arabidopsis T-DNA knockout lines	Arabidopsis Gene ID	Arabidopsis Gene
Clo-0	Wild	Wild
CS67959	AT1G08720	AtEDR1
SALK_088514C	AT5G12870	AtMYB46
SALK_151594	AT3G45640	AtMPK3-1
SALK_100993C	AT5G12870.1	AtMYB46
SALK_203382C	AT5G24530	AtDMR6
CS72352	AT5G45110, AT4G19660	AtNPR3, AtNPR4
SALK_025198C	AT3G56400	AtWRKY70
SALK_034157C	AT4G23810	AtWRKY53

Supplementary Table S2. ANOVA results for pairwise comparisons of mean values among RNAi-treated and control groups.

This table presents ANOVA results from pairwise comparisons of mean values in transient assays conducted on leaf and root-crown tissues. Experimental groups include RNAi constructs and the Empty control. Displayed metrics include mean values, quantiles, compact letter display for homogeneity grouping, mean differences with 95% confidence intervals, and adjusted p-values to indicate statistical significance.

Transient assay - Leaf

Group	Mean Value	Quantiles	Compact Letter Display	Mean Difference (Compared to Others)	Lower Bound (95% CI)	Upper Bound (95% CI)	Adjusted p-value
EV	17.8	23.6	b	-	-	-	-
EDR1-KD	37.9	52.4	a	Empty - RNAi_EDR1: -20.14	-32.24	-8.03	0.002

Transient assay- Root and Crown

Group	Mean Value	Quantiles	Compact Letter Display	Mean Difference (Compared to Others)	Lower Bound (95% CI)	Upper Bound (95% CI)	Adjusted p-value
EV	18.6	25.2	b	-	-	-	-
WAK1-KD	54.2	61.9	a	RNAi_WAK - Empty: 35.60	15.55	55.64	0.0011
CNGC1-KD	39.4	40	a	RNAi_WAK - RNAi_CNGC: 14.85	-6.08	35.79	0.1857
				Empty - RNAi_CNGC: -20.75	-40.79	-0.7	0.0423

Supplementary Table S3. Results of qRT-PCR analysis showing pairwise t-Test comparisons of mean expression levels between RNAi-treated and empty control groups.

Transient assay – Leaf

Group	Mean (expression level)	Standard Deviation (SD)	N	t-value	Degrees of Freedom (df)	p-value
EV	1.2365	0.254403	9	2.87	13	0.013*
EDR1-KD	0.2435	0.056547	6	-	-	-

Transient assay- Root and Crown

Group	Mean (expression level)	Standard Deviation (SD)	N	t-value	Degrees of Freedom (df)	p-value
EV	1.1255	0.208591	12	2.42	19	0.025*
WAK1-KD	0.6573	0.248651	9	-	-	-

Group	Mean (expression level)	Standard Deviation (SD)	N	t-value	Degrees of Freedom (df)	p-value
EV	1.1255	0.208591	12	2.11	22	0.047*
CNGC1-KD	0.7379	0.222176	12	-	-	-
